# Benzodiazepines: Uses, Dangers, and Clinical Considerations

**DOI:** 10.3390/neurolint13040059

**Published:** 2021-11-10

**Authors:** Amber N. Edinoff, Catherine A. Nix, Janice Hollier, Caroline E. Sagrera, Blake M. Delacroix, Tunde Abubakar, Elyse M. Cornett, Adam M. Kaye, Alan D. Kaye

**Affiliations:** 1 Department of Psychiatry and Behavioral Medicine, Louisiana State University Health Shreveport, Shreveport, LA 71103, USA; catherine.nix@lsuhs.edu (C.A.N.); janice.hollier@lsuhs.edu (J.H.); 2 School of Medicine, Louisiana State University Health Shreveport, Shreveport, LA 71103, USA; ces001@lsuhs.edu (C.E.S.); bmd001@lsuhs.edu (B.M.D.); taa001@lsuhs.edu (T.A.); 3 Department of Anesthesiology, Louisiana State University Health Shreveport, Shreveport, LA 71103, USA; elyse.bradley@lsuhs.edu (E.M.C.); alan.kaye@lsuhs.edu (A.D.K.); 4 Department of Pharmacy Practice, Thomas J. Long School of Pharmacy and Health Sciences, University of the Pacific, Stockton, CA 95211, USA; akaye@pacific.edu

**Keywords:** benzodiazepines, GABA, withdrawal, cognitive decline

## Abstract

Benzodiazepines (BZDs) are among one of the most widely prescribed drug classes in the United States. BZDs are a class of psychoactive drugs known for their depressant effect on the central nervous system (CNS). They quickly diffuse through the blood–brain barrier to affect the inhibitory neurotransmitter GABA and exert sedative effects. Related to their rapid onset and immediate symptom relief, BZDs are used for those struggling with sleep, anxiety, spasticity due to CNS pathology, muscle relaxation, and epilepsy. One of the debilitating side effects of BZDs is their addictive potential. The dependence on BZDs generally leads to withdrawal symptoms, requiring careful tapering of the medication when prescribed. Regular use of BZDs has been shown to cause severe, harmful psychological and physical dependence, leading to withdrawal symptoms similar to that of alcohol withdrawal. Some of these withdrawal symptoms can be life threatening. The current treatment for withdrawal is through tapering with clonazepam. Many drugs have been tested as a treatment for withdrawal, with few proving efficacious in randomized control trials. Future research is warranted for further exploration into alternative methods of treating BZD withdrawal. This call to action proves especially relevant, as those seeking treatment for BZD dependence and withdrawal are on the rise in the United States.

## 1. Introduction

Benzodiazepines (BZDs) are among one of the most widely prescribed drug classes in the United States. BZDs are a class of psychoactive drugs known for their depressant effect on the central nervous system (CNS). They quickly diffuse through the blood–brain barrier to affect the inhibitory neurotransmitter GABA and exert sedative effects. GABA is the most common neurotransmitter in the CNS, and BZDs primarily work on the GABA-A receptor subunit [1]. The GABA-A receptor has various subunits, and the most important in this case is the alpha (A) submit unit. The alpha subunit has various isoforms, which dictate a BZD’s effects on the CNS. The A1 subunit is believed to be responsible for the sedative effects and anterograde amnesia, and some of the anticonvulsive impacts of diazepam [1]. The A2 subunit isoform mediates the anxiolytic and myorelaxant effects [1]. In total, there are six isoforms of the GABA-A subunit. To be specific in terms of binding sites and actions, BZD binds between the alpha and the gamma subunit. It enhances the effect of GABA at the GABA-A receptor, allowing it to exert a more significant effect [2].

Given their lipid solubility, BZDs have a high volume of distribution in the body, which translates to higher tissue concentrations than blood. After exerting their effect, BZDs are metabolized primarily by the liver and excreted by conjugation, so they should be used in caution in the elderly, smokers, and those with liver disease or damage [3]. Due to their rapid onset and immediate symptom relief, BZDs are used for those struggling with sleep, anxiety, spasticity due to CNS pathology, muscle relaxation, and epilepsy. Their sedative effect aids in sleep and insomnia disorders by reducing sleep onset latency. Their CNS depressant effects potently reduce anxiety and abort acute-onset panic and anxiety attacks [4]. Benzodiazepines are also incredibly effective at rapidly aborting convulsant activity in those with epilepsy or other seizure disorders [5].

BZDs gained popularity in the 1960s and 1970s through household names such as The Rolling Stones and numerous Hollywood movies sensationalizing Valium (diazepam). BZDs were encouraged for anyone wanting to calm their nerves and ease their sleep, causing them to rapidly attain favor in society [6]. Additionally, given the continual rise of anxiety and sleep-disordered problems over the decades, BZDs remain a regular fixture in the United States today [7]. However, with this ongoing, widespread use comes the dark reality of BZD dependence [6].

Chronic users of the drug class often exhibit reliance with a high risk for abuse. This should provide pause, as BZD discontinuation after chronic use leads to withdrawal symptoms, with heavy users at risk for seizure activity [8]. Short-acting BZDs appear to carry more significant risks of adverse effects on abrupt cessation and usually exhibit greater dependence [9]. Several factors determine the rate and severity of withdrawal from the hypnotic. These are (1) the duration of use, (2) elimination half-life (short- or long-acting), (3) daily dosage, (4) rate of the taper and (5) the potency of the BZD itself [10]. One study found a withdrawal rate of approximately 40% in those using for six or more months with abrupt cessation of long-acting BZDs [10]. BZDs should especially be cautioned for use in the elderly, as the medication can lower the seizure threshold, cause gait instability, and balance problems [11]. Currently, the number of individuals seeking treatment for complications related to BZD use dependence is rising [5]. Therefore, the aim of this review is to highlight the indications for BZD use, the dangers of withdrawal from BZD, concerns regarding the cognitive decline associated with BZD use, and the clinical studies regarding this cognitive decline and treatment of withdrawal symptoms.

### 1.1. Indications for Benzodiazepines

BZDs are most commonly used for panic disorder and generalized anxiety disorder (GAD) regarding its indications for anxiety. Specifically, temazepam is commonly used for insomnia, clonazepam is commonly used for anxiety and seizures, lorazepam is commonly used for catatonia and seizure abortion when used intramuscularly or intravascularly, and diazepam is commonly used for anxiety, muscle spasms and rectally for seizures. In terms of anxiety, BZDs are used as a bridge when starting another medication or as abortive therapy for panic attacks. Given concerns for use dependence and withdrawal, SSRIs and antidepressants have been made mainstay therapy for these conditions. However, given their delayed onset of action, BZDs continue to be widely prescribed for these illnesses [12].

Other important indications for the use of BZDs include the treatment of catatonia, seizure disorders, and alcohol and BZD withdrawal. The drug class is the mainstay of treatment for catatonia, which is characterized by postural rigidity, immobility, purposeless activity, and disturbances in one’s consciousness [10]. Catatonia presents in populations suffering from bipolar disorder, schizophrenia, or a variety of medical conditions. BZDs act on the CNS to exert anxiolytic and sedative effects and, together with electroconvulsive therapy, are the mainstay therapy for catatonia [13].

BZDs are also used in urgent seizure emergencies, including status epilepticus, acute seizures, cluster seizures, and seizures due to alcohol and BZD withdrawal. Neuronal damage may begin as soon as five min of seizure activity, so abortion with BZDs or other CNS sedatives is imperative [14]. Diazepam, lorazepam, and midazolam are most widely used for abortion of prolonged seizures, as they all exert anticonvulsive effects when bound to the alpha 1, 2, and 5 subunits of the GABA-A receptor in the CNS. Alcohol withdrawal abruptly removes the previous, chronic sedative effect of alcohol on the CNS due to prolonged stimulation of GABA receptors, resulting in unchecked autonomic activity and increased sympathetic stimulation. This mechanism results in symptoms and signs of psychomotor agitation such as diaphoresis, anxiety, nausea, vomiting, tremors, and possibly seizures and delirium. BZDs bind to the effect of the inhibitory neurotransmitter GABA to slow the firing of the autonomic nervous system, preventing hyperstimulation and associated sequelae of withdrawal.

Long-acting BZDs are also used to taper, and eventually discontinue, short-acting, chronic BZD usage. Like alcohol, abrupt withdrawal of the sedative action of BZDs on GABA causes hyperstimulation of the autonomic nervous system with similar associated symptoms and signs. Long-acting BZDs are a component of mainstay therapy to prevent BZD withdrawals seizures [15].

### 1.2. Duration of Treatment

BZDs are extremely effective at relieving their indicated conditions. Still, due to the severe potential for dependence and deadly withdrawal sequelae, guidelines for recommended use are no longer than a few weeks. Despite this, numerous studies report usage extending for months into years or even decades in many users [9]. Additionally, according to several studies, BZD use increases with age, with long-term usage most prevalent in the 65 and older population [16]. Long-term use is defined as two or more months at a therapeutic dose and when used long-term, BZDs pose potential harmful effects. This is especially concerning in the elderly, who are at increased risk for psychomotor impairment, car accidents, and cognitive impairment such as anterograde amnesia-diminished short-term recall, and increased forgetfulness [11]. Some additional side effects of concern include aggressive behavior and expressing anger towards others in between 1% and 20% of users [17]. The main driving factor for dependence is the development of tolerance, causing users to need increasing doses for the same symptom relief [18].

## 2. Benzodiazepine Overuse Pathology, Misuses, and Complications

### 2.1. The Effects of Benzodiazepine on Cognition

Many studies have examined the effects of benzodiazepines on cognition with varied results. Some of the variations in results may be attributed to cognitive criteria in which each study followed. In a prospective longitudinal study on the effects of psychotropic drugs in the cognition of the elderly, Allard et al. [19] found no significant long-term effect of BZD use on cognition. This is supported in a similar study of elderly adults on a BZD for six months with drug discontinuation one month later. The study found no significant cognitive impairment in adults with long-term use of BZD [20]. A study of over 2000 older adults assessed the effects of chronic BZD use on cognition [21]. Chronic use of BZD leads to a small but significant change in fluid intelligence, while long-term use of BZD correlates with worse cognitive decline when compared to the effects of using a high dosage [21].

### 2.2. Side Effects of Benzodiazepine Use

BZDs have quite a few and significant side effect profiles. The risk of falls leading to injuries in elderly BZD users is significantly increased in patients greater than 80 years old, while the increased risk is not significant in patients under 80 [22]. Passaro et al. described an increased risk of falls in elderly hospitalized patients prescribed short-acting BZD [23]. For mothers with BZD use during pregnancy, there is a risk of premature birth and low birth weight. While the study showed some teratogenic effects of BZDs on fetuses, the result is not statistically significant, and some of the malformations seen in fetuses in the study may have been due to the use of other medications such as antidepressants [24]. One of the debilitating side effects of BZDs is their addictive potential. Their relative safety compared to fellow depressants or barbiturates have increased the rate at which they are prescribed [25]. The dependence on BZDs generally leads to withdrawal symptoms, which necessitates careful tapering of the medication when prescribed [26].

### 2.3. Misuse of Benzodiazepine

There has been a steady increase in the number of prescribed BZDs in the US [27]. Bachhuber et al. 2016 reported an increase in ED visits for overdose and increased overdose death due to BZD use [28]. Women are more susceptible to BZD overuse because they are more likely to be prescribed than men [29]. Mclean et al. 2011 reported that the diagnosis of anxiety and stress disorders has a higher prevalence in women, which can explain the discrepancies in the prescription for men vs. women [30].

It has been shown that patients diagnosed with substance use disorder have increased tendencies to misuse BZD compared to the general population [31]. Although women are prescribed BZDs more commonly than men, Mchugh et al., 2021 reported no gender-based misuse of BZD in adults with substance abuse disorder [32]. Most of the participants in the study reported coping as a reason for their misuse of BZD prescriptions. Cook et al. also supported the conclusion that there is no gender-based difference in BZD misuse, although their study found that white race is more likely than other races (Black, Hispanic and Asians) to misuse BZD due to the prevalence of more prescriptions given to white patients than others [33]. An additional study on BZD misuse in adolescents found that factors such as white race, lower family income, older age and delinquencies are associated with BZD misuse. At the same time, it also found a correlation between misuse and depression [34].

### 2.4. Complications of Benzodiazepine Abuse

One of the main categories of people with BZD prescriptions is those with insomnia. Manconi et al. explored the effects of long-term BZD use on sleep architecture and microstructure in those with insomnia. They found significant changes in sleep microstructure in chronic insomnia with high dosage abuse of BZD, but sleep architecture changes were not significant. Long-term use of BZD leads to negative changes in sleep microstructure in patients with insomnia [35].

A study of patients undergoing total knee arthroplasty reported that patients who have filled a BZD prescription within 6 months before their surgery are at a high risk of revision of the surgery and femur fracture fixation [36]. BZD use is associated with an increased risk of falls, which may explain the revisions. The study also found a correlation between patients who use BZD preoperatively and those who have postoperative delirium [36].

Several case reports have also linked tapering of different BZD with Takotsubo cardiomyopathy (TCM). Hashm et al. reported a case of TCM 2–3 days after starting the tapering of alprazolam in a 63-year-old Caucasian female which resolved after the previous dosage was restarted [37]. TCM has also been associated with the tapering of other BZD such as clonazepam and lorazepam [38,39].

### 2.5. Issues with Clinical Use of Benzodiazepine

In 2018, between 8.3% and 12.8% of BZD users in Switzerland have prescriptions from multiple physicians which resulted in the inability to track the number of prescriptions a patient is given yearly [40]. In a survey of British general practitioners, many reported pressures in prescribing BZD to patients and a lack of adequate knowledge on alternative psychological treatment for insomnia [41].

BZDs are classified either by their duration of action or potency. The difference in these characteristics dictates the clinical applicability of the drugs. Oxazepam, temazepam, and chlordiazepoxide which are low potency benzodiazepines are well tolerated with low toxicity levels. Alprazolam, lorazepam and clonazepam are high potently clinically used to treat panic disorders and serve as adjuncts for treating many other diseases [1]. Due to their toxic effect on the central nervous system, appropriate care is necessary with BZD. BZDs lead to long-lasting impairment of episodic implicit memory while it only impairs implicit memory transiently [1]. They also lead to disinhibition, impairing the user’s ability to appropriately assess the risky actions or behaviors. Elderly patients in intensive care can develop delirium if they are on a BZD [1].

Researchers studied the neuroplastic effects of diazepam on mice. Results showed that, compared to wild-type mice, mice on diazepam experienced longer uninterrupted sleep [42]. It also reduces the expression of mRNA transcripts such as CaMKIIa, BDNF, GIF, c-fos, NGFIa which are necessary for regulating synapses and plasticity [42]. The suppression of CaMKIIa by diazepam has a long-lasting effect leading to a limited neuronal response to changes in intracellular calcium and decreased response by GABA-A receptors [42].

## 3. Benzodiazepine Use Dangers

BZDs are one of the most prescribed drugs in the US. BZDs are a common cause of non-opioid overdose. Agarwal and Landon 2019 stated that the prescription of BZD in outpatient settings significantly increased from 2003 to 2015 [27]. Several studies have examined the relationship between BZD use and mortality. In a systematic review of research, Charlson et al. could not find conclusive evidence supporting increased risk of mortality in BZD users [43]. In their analysis of six retrospective studies, half of them showed no significant relationship between BZD use and mortality. One of the studies showed an increase in mortality with increased dosage and frequency of use which could be attributed to increased toxicity, another showed a positive correlation among older adults [43]. One of the study’s limitations is the inability to collect data that differentiate mortality correlation with illicit BZD use from the use of prescribed pills.

A study analyzing over 1000 cases of oxycodone-related drug abuse deaths showed that BZDs are among the most abused drugs by individuals using multiple drugs of abuse. The study showed that in oxycodone users, combination use with diazepam was the most prevalent [44]. This was further supported by data from Rooney et al. which reported that 54% of abusers of oxycodone are also dependent on a BZD [45]. The study also showed that 64% of heroin users are also abusing BZD [46].

Researchers investigating the correlation between anxiolytic and hypnotic drugs with mortality hazards examined over 100,000 patients in a retrospective cohort study. They found that the hazard of death was doubled in patients prescribed BZD compared to control patients [47]. The result was statistically significant. There was an association between the prescription of anxiolytic drugs and mortality, resulting in 4 excess deaths in the anxiolytic drug group within an average of 7.6 years [47]. The relationship between hypnotics and cancer was expanded upon by Kripke et al. They found a 35% increased chance of developing a new non-melanoma cancer in users of hypnotics [48]. In agreeing with some of the studies above, there was also a 4.6-fold increase in the hazard of death in patients on hypnotics over 2.5 years [48]. This is more significant than the study by Weich et al. [47] which found a 2-fold increased hazard of death in 7 years. It was also reported that the risk of death in patients using less than 18 pills per year is increased by 3.6-fold [48].

Abrahamsson et al. investigated the relationship between hypnotic drug overdose versus non-overdose deaths in patients on opioid maintenance therapy. They showed that benzodiazepine increased the incidence of non-overdose death in these patients which may be attributed to its impairment of cognition, sensory, and motor skills and increased risk of fall leading to injuries [49]. Despite the increase in risk, less than 13% of the non-overdose deaths were trauma related. A study investigating the prescription of opioids and BZD in veterans showed an increased risk of overdose death in veterans who have prescriptions for both BZD and opioids at the same time [50]. The dose of BZD given also positively correlated with increased risk of an overdose death [50].

In a literature review exploring the relationship between BZD and suicide risk, it stated that although anxiety and insomnia which are often treated with a BZD, it can increase the risk of suicide; several studies in the literature have suggested that BZD use is also involved in increased suicide risk in its users [51]. The use and discontinuation of alprazolam within 2 weeks disrupt sleep onset and quality, increasing suicide risks [51].

## 4. Withdrawal

### 4.1. Symptoms of Withdrawal

Regular use of BZDs has been shown to cause serious, harmful psychological and physical dependence, leading to withdrawal symptoms similar to that of alcohol withdrawal. Regular use of BZDs can lead to tolerance, which is the physiologic dependence on the presence of BZDs in the body’s system. This can be linked to addiction as the patient is not just psychologically addicted to the substance, which can be seen with cravings, and physical addiction. Withdrawal, like with alcohol since they exert their effects on similar receptors, can be life threatening. Withdrawal occurs as the BZD concentration in blood and tissue declines, generally causing symptoms opposite to that of the drug’s therapeutic effects. Psychological symptoms include increased excitability, nightmares, anxiety, insomnia, panic attacks, depression, hallucinations, irritability, paranoid thoughts, social phobia, poor memory, poor concentration, delirium, and even psychosis. Physical symptoms include headache, seizures, pain or stiffness in the head and neck region, an altered sensation of limbs, weakness and fatigue, tingling and numbness, muscle twitches, tremors, gastrointestinal symptoms (abdominal distension, nausea, diarrhea, constipation, etc.), appetite and weight changes, unusual smell, and others have been documented [52]. These symptoms may last for one to a few weeks after cessation, with duration and severity largely depending upon the amount of time spent chronically taking the BZD, the half-life of the specific BZD, and the daily dose consumed [26].

### 4.2. Mechanism of Withdrawal

Symptoms associated with withdrawal occur due to the chronic effect on the GABA-A receptors in the CNS to BZDs. Over time, this causes a neuroadaptive process of both desensitization of the inhibitory function of GABA, sensitization of excitatory glutamine receptors, and possibly sensitization of N-methyl-D-aspartate (NMDA) receptors, along with other receptors. These changes contribute to tolerance to specific BZD and dosage, mediating dependence and later withdrawal [53].

### 4.3. Alprazolam

One of the most well-studied BZDs in the setting of withdrawal is alprazolam. Due to its short half-life, and rapid absorption, alprazolam is distinguished as one of the most rapid-acting BZD with fastest relief of symptomology, increasing its abuse liability [54]. Alprazolam is widely used as monotherapy for panic disorder and anxiety and was found superior to other forms of monotherapy for these conditions including other BZD, non-SSRI antidepressants, and buspirone. This superior effect is thought to be due to its unique alpha-2 adrenergic activity, enhancing its potency for relieving panic and anxiety disorders. This same mechanism is also thought to be the cause behind alprazolam’s strong rebound hyperadrenergic effects with cessation [54,55]. Many drug therapies have been suggested as treatment for alprazolam withdrawal with few rendered effective. These treatments will be discussed briefly below.

### 4.4. Special Populations and Withdrawal

#### 4.4.1. The Elderly

Withdrawal has potential to be ubiquitously dangerous, but there are four main populations susceptible to the potentially life-threatening dangers of BZD dependence. First, the elderly (over 65 years old) are of extremely high risk due to their predisposition for falls, confusion, insomnia, memory loss, and other psychiatric problems regardless of BZD use. As the brain changes with age, the regions of the brain responsible for various sleep patterns and behaviors shrink, often resulting in less restful sleep, inability to sleep for longer durations, and fewer sleep spindles generation during non-REM sleep, a process necessary for memory consolidation and new information retention [56]. Because of these sleep changes and their predisposition for insomnia, the elderly may more often have chronic BZD use for sleep relief. However, given BZD’s potential side effects of psychomotor retardation, amnesia, and increased forgetfulness, their use in the elderly is incredibly dangerous. Cessation after long-term usage (greater than two weeks) has even shown in some individual’s the inability to return to cognitive baseline. It has been associated with increased risk for dementia [57,58]. The elderly also have a decreased body water concentration, causing usual doses to elevate to potentially toxic levels quickly, exacerbating all effects, negative and otherwise, of BZDs. Rarely, catatonia and delirium can develop as part of the withdrawal syndrome. Given this picture, withdrawal symptoms should be carefully treated with BZD long-acting taper, with decreasing doses slowly over time, along with psychotherapeutic interventions such as motivational enhancement and cognitive behavioral therapy (CBT) with psychoeducation [59].

#### 4.4.2. Children

Secondly, approximately 20% of the children in the intensive care unit given BZD during sedation, more specifically midazolam, have been shown to exhibit withdrawal effects. The severity of the withdrawal sequelae depends on the total dose and duration of infusion and usually presents as agitation, tremors, difficulty sleeping, and inconsolable crying [60].

#### 4.4.3. Pregnant Women and the Fetus

Pregnant women and fetuses are at increased risk for adverse effects of withdrawal; they both metabolize BZD slowly, and the drug can cross the placenta to cause concentrations to build up to significant levels in the neonate [18]. While a therapeutic dose has not been proven teratogenic, use during pregnancy has been linked to low birth weight, preterm labor, and intrauterine growth restriction. The unborn fetus is at high risk for “floppy infant syndrome,” characterized by muscle laxity, failure to suckle, and oversedation. Approximately two weeks after birth, the infant experiences withdrawal consisting of continued difficulty feeding, high pitched cries, hyperexcitability, and consequently possible failure to thrive. The ultimate concern is that such fetuses will later be susceptible to autism, learning difficulties, attention deficit disorder, and general hyperactivity [24].

### 4.5. Treatment for Benzodiazepine Withdrawal

There are pharmacological options for treatment in those suffering from withdrawal or wishing to discontinue their chronic BZD use. Many alternate drug therapies have been proposed to reduce the severity of withdrawal, including alpha-blockers (propranolol and clonidine), anticonvulsants (valproic acid, lamotrigine, carbamazepine, and phenobarbital), progesterone, baclofen, and trazodone. Each of these studies received mixed results, with no statistically significant advantage to BZD therapy. The current treatment of choice is to switch the current short-acting BZD for a long-acting alternative then gradually taper the dose to wean the individual off BZD completely [8]. Clonazepam has been used in the outpatient setting as a medication for taping the use of BZD. However, no set schedule for a taper has been validated in the current literature.

## 5. Clinical Studies

Many clinical studies have been conducted to assess the severity and treatment of withdrawal systems, while others assess more long-term effects of chronic BZD use. The mainstay of BZD withdrawal treatment at this time is a slow taper off the drug to prevent severe withdrawal symptoms; however, many patients cannot tolerate this taper without experiencing rebound anxiety and other symptoms. Current studies are aimed to decrease this rebound anxiety effect while also decreasing relapse into BZD use using different medications, counseling, BZD dosing strategies, or different tapering techniques.

### 5.1. Factors Influencing Withdrawal Symptoms

Certain factors play a role in the severity of withdrawal symptoms, such as dosage, duration of action of the BZD duration of treatment with the drug, and severity of psychiatric symptoms pre-treatment. Studies have shown that treatment for longer periods with high-dosage, short-acting BZD contribute to more severe withdrawal effects [61]. Milder effects are seen with longer-acting BZD that are used for shorter periods [61]. Those who have never experienced withdrawal symptoms from BZD discontinuation could quit using BZD more easily [62]. Baseline anxiety is an important indicator of withdrawal symptoms [63]. People with severe anxiety before starting treatment with BZD typically have more severe withdrawal symptoms, and thus have a harder time fully discontinuing the drug [63]. Psychiatric diagnoses have also been linked to one’s ability to discontinue treatment with BZD. One study showed a high co-occurrence with BZD dependence and all psychiatric disorders in general [64,65]. Specifically, those with cluster B personality disorders have the worst prognosis in regard to discontinuing BZD. In one study, not a single subject diagnosed with a cluster B personality disorder successfully discontinued BZD use [63]. They also had a higher dropout rate from the study [63]. Additionally, younger patients tend to have a decreased success rate of discontinuing BZD use than older patients [66]. Interestingly, those who used alcohol while taking BZD experienced no difference in discontinuation rate from those who did not use alcohol [64].

### 5.2. Pharmacologic Management of Withdrawal Symptoms

Many medications have been tested to alleviate withdrawal symptoms and make it easier for patients to discontinue BZD since a gradual taper does not always lead to successful discontinuation of the drug. Currently, a gradual taper with clonazepam is used as maintenance therapy for BZD-dependent patients. However, it still carries the risk for abuse and dependence since this is also a BZD, albeit a slow-acting one [67].

One medication that is not a BZD that has been used to treat BZD withdrawals is flumazenil. This medication is currently FDA approved for BZD overdose. Flumazenil is a BZD antagonist, which works by displacing BZD from its binding site without having an effect on GABA itself. IV infusion of this medication has been shown to decrease aggression during BZD withdrawals by self-report in one study [68]. One study showed that a 7 day IV infusion of flumazenil during withdrawal in patients stabilized with clonazepam or antidepressants led to abstinence in over half of their subjects [67]. However, in the same study, for those with polysubstance abuse, flumazenil did not reduce cravings for other drugs and did not restore BZD receptors’ sensitivity [67]. This study was limited by the small sample size, a lack of double-blinding, multiple parallel treatments, and had the potential for subjects to fail to report relapse in drug use [67]. More careful research will need to be performed in this area. Some researchers are even looking into long-term subcutaneous administration of flumazenil for BZD-dependent patients. This could be a feasible alternative to IV infusion of flumazenil because patients can be mobile when given medications subcutaneously, so they are more likely to return for repeat treatments and recommend treatment to friends [64]. It has been shown that the efficacy of withdrawal treatment might be more associated with levels of flumazenil in cerebral spinal fluid rather than plasma levels of the drug [64]. Clinical Institute Withdrawal Assessment Alcohol Scale (CIWA) symptoms peaked at day 1 and decreased significantly during days 2 and 3, leading researchers to this conclusion in one study [64]. However, Flumazenil carries a high risk of seizures, so one must be cautious when administering this drug [68]. Flumazenil is continuously shown to be a viable option for BZD withdrawal treatment; however, other medications currently on the market might be more efficacious.

The beta-blocker propranolol has shown mixed results when it comes to treating BZD withdrawal and dependence. One study found that propranolol attenuated some withdrawal symptoms in patients who stopped taking either diazepam (a long-acting BZD) and those who took lorazepam (a short-acting one) abruptly [69]. However, in the same study, 27–45% of patients experienced withdrawal symptoms even while taking propranolol [69]. The control group and the experimental group had the same dropout rate in this study [69]. Another study found that abrupt discontinuation and daily administration of propranolol in severely dependent patients was not a more successful treatment plan than current practice [65]. Additionally, in this study, approximately 80% of patients experience withdrawal symptoms, which is much greater than the rate in other studies [65]. The authors attribute this to the severity of patients’ dependence on BZD before treatment with propranolol [65]. More studies need to be performed on treating withdrawal with propranolol, including testing it as a potential adjunct to tapering off both long-acting and short-acting BZD.

Captodiamine is a diphenhydramine-related compound that does not work at histamine receptors as diphenhydramine does and its mechanism of action is unclear [70]. This drug has also been studied in the context of both BZD replacement and withdrawal as a potential treatment [70]. One study showed that replacing BZD with a 45 day captodiamine led to a decrease in severity of withdrawal symptoms in patients taking BZD for six months [70]. Another interesting finding was that after the discontinuation of captodamine treatment, there was no emergence of withdrawal symptoms, suggesting that captodiamine might have a different mechanism of anxiolysis than BZD [70]. Additionally, during captodiamine treatment, psychomotor function improved in all areas tested from beginning to end of treatment [70]. It must be noted that these patients were taking relatively low doses of BZD pre-treatment [70]. Captodiamine is showing promise as a potential medication for the management of BZD withdrawal syndrome; however, more research needs to be performed on the side effects and safety profile of the drug.

The antiepileptic oxcarbazepine has also shown potential to ameliorate withdrawal symptoms more than older-generation antiepileptics such as carbamazepine [71]. Oxcarbazepine has a better side effect profile and is a more tolerable anticonvulsant than older antiepileptic drugs [71]. One case series showed that not only did oxcarbazepine provide more tolerability than the current mainstay of treatment, but it also shortened the withdrawal time frame for patients to just 11 to 19 days, even for patients previously taking high doses of BZD [71]. It is important to note that this study was uncontrolled, so further randomized controlled studies need to be performed to increase the validity of these results [71].

Benzodiazepine abuse is common in those on methadone maintenance treatment (MMT), so special consideration must be taken for those withdrawing from the drugs while on MMT [68]. These patients are more likely to die from methadone toxicity because of the synergistic effects of methadone and BZD [68]. Additionally, these patients are more likely to have comorbid substance use disorders and anxiety disorders so it can be harder to find an efficacious treatment for their withdrawal symptoms [68]. One potential candidate for treatment of withdrawal symptoms in these patients is gabapentin, which works similarly to the neurotransmitter GABA [68]. However, one study showed no significant difference in BZD use in MMT patients between gabapentin and placebo [68]. However, this study was limited by a small sample size, so further randomized clinical trials need to be conducted to assess the efficacy of gabapentin treatment in BZD -dependent MMT patients [68]. Further studies need to be performed on not just gabapentin, but other medications for MMT patients with BZD dependence should be evaluated since treating them is more complicated.

### 5.3. Educational Approaches to Benzodiazepine Withdrawal

Other studies have assessed different methods of counseling on BZD dangers and alternatives to patients alongside a gradual taper off the drugs. One study compared the mainstay of treatment with a standardized interview/counselling approach to treatment [72]. The experimental group in this study had a weekly 1/10-dose reduction after a 2 week stabilization period [72]. The experimental treatment also included a BZD diary, a drinking diary, BZD withdrawal education, and assessments for ways of coping and “progressive relaxation exercise” [72]. This was compared to a gradual taper without the other components of the treatment plan [72]. There was no difference in the success rate of BZD discontinuation between the control and experimental groups in this case [72].

Another study that tested a different standardized education protocol showed more promising results [73]. The experimental group in this study was counseled on the first visit for 15–20 min on the effects, dangers, and alternatives to chronic BZD use and dependence [73]. The subjects were interviewed with surgery-based consultations for approximately 10 min [12]. This study found that patients undergoing this structured intervention were 5-fold more likely to successfully discontinue BZD than those who just tapered off the drug [73]. Interestingly, a lower prevalence of withdrawal symptoms was noted in the experimental group without any change in pharmacologic treatment from control group [73]. However, this study included a small sample size, so a larger study using this standardized counseling method would increase the validity of the results of this study [73]. More studies will need to be carried out on the non-pharmacologic treatment of BZD withdrawal, as it is showing some promise for the successful discontinuation of the drugs.

### 5.4. Long-Term Effects of Benzodiazepine Use

Some studies in the past have shown that there is a correlation between chronic BZD use and a decline in cognitive function, including the development of dementia and dementia-like diseases. One study showed a potential for cognitive decline after BZD use in the elderly, but at the same time did not find a link between their use and the development of Alzheimer’s dementia [73]. The researchers in the study cautioned the prescription of BZD in the elderly due to the potential for cognitive decline [73].

Other studies have shown that there is no correlation between BZD use and cognitive decline. One study showed that administration of BZD in patients with Alzheimer’s disease do not lead to further cognitive decline after 18 months of taking the drug [74]. The subjects in this study had mild to moderate Alzheimer’s dementia and showed no change in AD-Cog scores after treatment with BZD [74]. Researchers in this study also cautioned, however, that these drugs are known to cause delirium, falls, and other adverse events in the elderly, so when possible, prescribers should either abstain from prescribing BZD to elderly patients or deprescribe them when possible [74]. Additionally, BZD have been shown not to increase or decrease Mini Mental Status Exam (MMSE) scores, caregiver burden (CB), or the Neuropsychiatric Inventory (NPI) in Alzheimer’s patients over 12 months of treatment in one cohort study [75]. Interestingly, in this same study, SSRIs and atypical antipsychotics showed the same results; however, trazodone actually improved the NPI [75].

So, while some more recent studies have shown that BZDs do not affect cognitive function, prescribers should proceed with caution when prescribing these drugs to elderly patients and dementia patients because of the risk of delirium and falls in this patient population. Table 1 summarizes the studies discussed in this section.

## 6. Conclusions

BZDs represent one of the most widely prescribed drug classes in the United States. They are used for immediate symptom relief of anxiety, epilepsy and other seizure disorders, spasticity from CNS pathology, catatonia, sleep disorders such as insomnia, and withdrawal from alcohol and other BZDs [3]. Chronic use of BZDs has been linked to a decline in cognitive function, increased risk of dementia and dementia-like illnesses, and impaired sensory and motor function in the elderly, as well as aggressive behavior and expressive anger in a subset of consumers [15,75].

BZDs show an increased risk for abuse and dependence. Over time, the inhibitory function of GABA may be desensitized with a corresponding sensitization of excitatory glutamine receptors. This neuroadaptive process builds tolerance, causing many chronic users to need increasing dosages for similar effects. As the drug concentration in blood and tissue decline, the sympathetic nervous system, possible seizures, and generally symptoms opposite that of its intended, therapeutic effect. This is of chief concern in four main populations: the elderly, children, pregnant women, and their neonates, given their sensitivity to BZD effects. One study further showed that the risk of dying is doubled in patients prescribed BZD compared with controls [47].

Current treatment for withdrawal is through tapering with clonazepam, and overdose should be treated with flumazenil [67]. Many drugs have been tested as a treatment for withdrawal, with few proving efficacious in randomized control trials. There is room in the research body for further exploration into alternative methods of treating withdrawal that does not include the BZD itself. This call to action proves especially relevant, as those seeking treatment for BZD dependence and withdrawal are on the rise in the United States [3].

## Figures and Tables

**Table 1:** Descriptions of studies performed on specific treatments of benzodiazepine dependence and withdrawal symptoms.

Treatment	Studies
Flumazenil	IV infusion of flumazenil over 7 days during withdrawal period, patients stabilized with clonazepam or antidepressants. An improvement in abstinence was found Self-reported effects of flumazenil on aggression during withdrawals. A self-reported decrease in aggression in patients was found Comparison of IV infusion to subcutaneous administration of flumazenil. Subcutaneous administration was found to be more tolerable for patients
Propranolol	Abrupt discontinuation of benzodiazepines in severely dependent patients and administration of propranolol rather than a slow taper. Researchers found no difference from mainstay treatment [66] Propranolol administration to patients after abrupt discontinuation of diazepam and lorazepam. Researchers found some difference from mainstay treatment
Captodiamine	Administration of captodiamine to benzodiazepine-dependent patients for 45 days after abrupt cessation of benzodiazepines. Researchers found no withdrawal symptoms present after discontinuation of captodiamine
Anticonvulsants	A case series on patients taking oxcarbazepine after cessation of benzodiazepines. A shorter withdrawal period was noted
Antipsychotics	A comparison of cyamemazine to bromazepam after 3 month treatment with benzodiazepines. Cyamemazine was found to be as effective as bromazepam in treating withdrawal symptoms
Standardized counselling protocols	Comparison of a slow taper to counselling on the dangers of benzodiazepines and alternatives to treatment. Researchers found no difference in treatments Comparison of a slow taper to a standardized interview and education protocol alongside a slow taper. Researchers found a significant improvement in symptoms and success rate in benzodiazepine discontinuation in the experimental group

## Data Availability

Data supporting the results above can be found on PubMed.
